# Physiological indicators of habitat quality for a migratory songbird breeding in a forest invaded by non-native Japanese barberry (*Berberis thunbergii*)

**DOI:** 10.1093/conphys/coaa037

**Published:** 2020-05-03

**Authors:** Chad L Seewagen, Eric J Slayton, Susan Smith Pagano

**Affiliations:** 1 Great Hollow Nature Preserve & Ecological Research Center, 225 State Route 37, New Fairfield, CT 06812, USA; 2 Thomas H. Gosnell School of Life Sciences, Rochester Institute of Technology, 84 Lomb Memorial Drive, Rochester, NY 14623, USA

**Keywords:** condition indices, forest arthropods, invasive species, ovenbird, seiurus aurocapilla

## Abstract

Non-native, invasive plants can impact birds by altering food sources, nesting substrates and other critical resources. Japanese barberry (*Berberis thunbergii*) is one of the most invasive, non-native woody plants in in the northeastern USA, and yet almost nothing is known about its effects on birds or other wildlife. To investigate individual-level impacts of Japanese barberry on a forest-breeding bird, we compared food abundance (leaf-litter arthropod biomass) and the physiological condition of territorial male ovenbirds (*Seiurus aurocapilla*) between areas of a forest preserve in New York State that had high or low densities of Japanese barberry. We used haemoglobin and plasma triglyceride concentrations to indicate energetic condition, plasma uric acid and total plasma protein levels to indicate diet quality, and heterophil to lymphocyte ratios to indicate chronic stress. We found no difference in arthropod biomass between ovenbird territories that were heavily invaded by or relatively free of Japanese barberry. Perhaps largely as a result, we found no relationship between Japanese barberry density and any of our five haematological condition indices. There was also no difference in body mass, body size or age ratio between ovenbirds nesting in areas with low or high densities of Japanese barberry to suggest that relatively uninvaded forest patches were in greater demand and acquired by the most dominant individuals. Our results indicate that Japanese barberry does not reduce habitat quality for breeding male ovenbirds in a way that affects their prey abundance or physiological condition, but we caution that other species of birds and other aspects of habitat quality could be affected differently. We encourage future research on additional bird species and the effects of Japanese barberry on factors such as diet composition, pairing and nesting success and post-fledging survival to improve science-based decision-making about the extent to which conservation resources should be applied towards Japanese barberry control.

## Teaser

We found no indication that Japanese barberry, a highly invasive, non-native shrub in north-eastern US forests, impacted nesting habitat quality for adult male ovenbirds in a way that reduced the abundance of their arthropod prey or their physiological condition.

## Introduction

Non-native, invasive plants and other biological invaders are a significant threat to global biodiversity and one of the greatest conservation challenges around the world (Wilcove *et al*., 1998; Mooney and Cleland, 2001). By outcompeting and diminishing the diversity of native plants in invaded habitats, one of the many ecological impacts that non-native, invasive plants can have is to simplify arthropod communities (Mitchell *et al.*, 2006; Litt *et al*., 2014) and trigger cascading impacts to birds and other consumers at higher trophic levels (Schirmel *et al*., 2016; Narango *et al*., 2018). The alterations to arthropod communities are driven by both aboveground interactions between non-native invasive plants and herbivores, and belowground changes in soil properties that influence soil and leaf-litter biota (Vestergard *et al*., 2015; McCary *et al*., 2016), thereby impacting the prey base of invertivores in multiple habitat strata.

Most temperate forest songbirds time their breeding activity to coincide with peak arthropod abundance in order to meet the high energetic demands of reproduction (Daan *et al*., 1989; Visser *et al*., 2004). Arthropod availability is a primary factor affecting the reproductive productivity of forest songbirds (Nagy and Holmes, 2004, 2005; Zanette *et al*., 2006), and any changes in arthropod community structure and decreases in abundance caused by non-native, invasive plants would therefore be likely to negatively impact bird fitness via reductions in clutch size, nestling quality and/or fledging success. Indeed, these indirect impacts of non-native plants on breeding birds through alterations of their arthropod prey base have recently been observed in Carolina chickadees (*Poecile carolinensis*), whose reproductive rate was found to be limited by the prevalence of non-native plants in residential yards in the Washington D.C. area (Narango *et al*., 2018), and common yellowthroats (*Geothlypis trichas*), whose nestling quality and growth rate were negatively affected by the prevalence of non-native plants in New Hampshire shrublands (Tarr, 2017). These and several additional studies (reviewed by Schirmel *et al*., 2016; Nelson *et al*., 2017) indicate that non-native, invasive plants pose significant threats to birds across individual, population and community levels.

Others have argued that non-native invasive plants can provide food, shelter or other resources that benefit birds, particularly in disturbed areas where native vegetation is less able to persist irrespective of the presence of non-native plants (reviewed by Schlaepfer *et al*., 2011). One frequently cited example is the current dependence of the federally endangered southwestern willow flycatcher (*Empidonax traillii extimus*) on non-native, invasive salt cedar trees (*Tamarix* spp.), which grow in riparian areas of the southwestern USA that are no longer suitable for native tree species that occurred there before damming significantly altered natural flooding regimes (Sogge *et al*., 2008; Stromberg *et al*., 2009; Schlaepfer *et al*., 2011). Cases like this complicate conservation action, and along with the substantial labour, cost and incidental impacts associated with large-scale invasive plant removal, necessitate an understanding of species-specific effects that non-native, invasive plants have on wildlife rather than basing management decisions on broad generalizations and assumptions (Nelson *et al*., 2017).

Japanese barberry (*Berberis thunbergii*) is a woody shrub first introduced to North America from Asia in the late 1800s and now one of most widespread alien plants in forests of the northeastern USA (Ehrenfeld, 1997; Silander and Klepeis, 1999). It causes changes in soil structure, chemistry, microbial communities and nutrient cycling that limit the growth of other woody plants and allow it to overwhelmingly dominate forest understories (Kourtev *et al*., 1999, 2002, 2003; Ehrenfeld *et al*., 2001; but see Flinn *et al*., 2014). Its stems and leaves are unpalatable to white-tailed deer (*Odocoileus virginianus*), which releases it from the browsing pressure that limits many native understory plants and gives Japanese barberry further competitive advantage (Harrington *et al*., 2004; Williams *et al*., 2009). Despite Japanese barberry’s spread throughout much of the forested northeastern USA, its effects on habitat quality for birds and other wildlife have received little attention and remain poorly understood.

Among many unknowns about the ecological impacts of Japanese barberry is whether it affects food availability and the physiological condition of birds that inhabit heavily invaded areas. While our previous work with a subset of the data collected for the present study suggests that the biomass of arthropods in the forest understory and leaf-litter layer—a primary food source for many birds—is unaffected by Japanese barberry, we found substantial differences in arthropod abundance and species composition between Japanese barberry and native vegetation that have the potential to affect both food availability and quality (Clark and Seewagen, 2019). This is particularly so for any bird species that favour or depend upon taxonomic groups that we found to be heavily reduced in barberry-invaded habitat, such as spiders and ants. Food availability and quality can influence the reproductive output of birds via their effects on the physiological condition of adults and the energetic resources they are able to allocate to egg production and parental care (Martin, 1987; Selman and Houston, 1996). Measures of food abundance paired with information on the physiological condition of the consumers can therefore provide a better indicator of food availability and quality than measures of food abundance alone. Physiological tools have so far been underutilized in studies of the impacts of invasive plants to wildlife despite their utility for determining the responses of individual animals to various forms of environmental change (Lennox *et al*., 2015; Nelson *et al*., 2017).

Here, we investigated differences in leaf-litter invertebrate biomass and the physiological condition of territorial male ovenbirds (*Seiurus aurocapilla*) between areas of forest with differing levels of Japanese barberry density to provide the first information on the relationship of this non-native plant with a bird’s physiological condition and associated prey base. We used haemoglobin (Hb) and plasma triglyceride (TRIG) concentrations to assess energetic condition, plasma uric acid (UA) and total plasma protein (TPP) levels to assess diet quality, and heterophil to lymphocyte ratios (H:L) to assess chronic stress levels of ovenbirds defending territories that were heavily invaded by or relatively free of Japanese barberry. If Japanese barberry creates poorer habitat conditions for ovenbirds, birds inhabiting areas with a high density of Japanese barberry would be expected to have lower levels of Hb, TRIG, UA and TPP, and higher H:L ratios than those whose territories are composed of mostly native vegetation.

## Materials and methods

### Study site

We conducted our study at Pawling Nature Reserve in the towns of Pawling and Dover, New York, USA (41.616669, −73.563227). The ∼430-ha reserve is predominantly composed of mature, second-growth oak and mixed hardwood-conifer forest. Additional habitat types include old fields, red maple swamps, vernal pools, freshwater streams and a lake. Some areas of Pawling Nature Reserve exhibit so-called invasion fronts (Kourtev *et al*., 2002; Maerz *et al*., 2009), where Japanese barberry is dense up to a point beyond which it is only sparse or still absent. Historical stone walls that remain from past agricultural uses of the land are also present in some parts of the preserve and divide areas that have different densities of Japanese barberry on each side, likely as a result of the type of former use (e.g. pasture vs. crops) and when the activity was most recently abandoned (DeGasperis and Motzkin, 2007). The variation in Japanese barberry density throughout our study site allowed us to compare leaf-litter arthropod biomass and the physiological condition of territory-holding birds between areas with different levels of invasion intensity while holding other primary habitat characteristics largely constant (*sensu*Maerz *et al*., 2009; Nuzzo *et al*., 2009; Clark and Seewagen, 2019).

### Study species

The ovenbird is a long-distance migratory songbird that breeds in mature hardwood and mixed forests throughout much of the northern USA and Canada, and overwinters in Mexico, Central America and the Caribbean islands. Ovenbirds are ground-nesting and ground-foraging invertivores that primarily feed upon and provision their young with a variety of arthropods found within leaf litter on the forest floor (Stenger, 1958; Holmes and Robinson, 1988). During the breeding season, males defend territories that typically vary in size from ~0.5–1.5 ha (Porneluzi *et al*., 2011), depending on food abundance (Stenger, 1958; Smith and Shugart, 1987). The ovenbird has been widely used as a model songbird to study impacts to breeding habitat quality from a variety of anthropogenic disturbances, such as timber harvesting, motor vehicle activity, energy infrastructure noise, large-scale agriculture and urban sprawl (Porneluzi *et al*., 2011), but we are unaware of any studies of the effects of invasive plants on ovenbirds.

### Bird capture

Territorial male ovenbirds were target-captured between 06:30 and 11:30 EST, 16–25 May (the primary nesting period of ovenbirds in the area), 2017 and 2018, using a 6-m mist-net and playback recording broadcast through a wireless speaker. We only attempted to capture ovenbirds that were heard consistently singing for several minutes from (i.e. defending) an area that we visually estimated, as a temporary measure, to have either little to no Japanese barberry (<10% of ground cover) or a relatively high density of Japanese barberry (>25% of ground cover) over an area of at least 0.5 ha in each direction and were at least 50 m in from the nearest forest edge. This was intended to ensure that Japanese barberry density was consistently low or high across the majority, if not all, of a bird’s territory, as ovenbird breeding territories are typically ~0.5–1.5 ha in size (Porneluzi *et al*., 2011). The aggressive responses of birds to the playback recording which resulted in their capture gave further assurance that we were within their territory. We returned to each capture location within 4 weeks to quantify shrub density and ground coverage with standardized survey methods in order to assign captured birds to low and high Japanese barberry density categories (see below). Within 5 min of capture, up to 150 μL of blood was collected by brachial venipuncture with a 26-gauge needle into a heparinized capillary tube and stored in the field in a cooler with ice packs. Birds were then banded with a US Geological Survey (USGS) aluminium leg band, measured (unflattened wing length to the nearest 1 mm), weighed (to the nearest 0.1 g on a digital balance), aged as second-year or after-second-year on the basis of rectrix shape and other plumage criteria, when possible (Donovan and Stanley, 1995; Pyle, 1997), and released. Bird capture and blood sampling was conducted under appropriate permits from the New York State Department of Environmental Conservation (License to Collect and Possess—Banding #114) and USGS (Bird Banding Permit #23755), and approved by the Rochester Institute of Technology’s Institutional Animal Care and Use Committee (proposals 2011-6, 2019-1).

### Vegetation sampling

For each captured bird, we measured shrub density and ground cover in a 0.02-ha circular plot that was centred on the midpoint of the mist-net in which the bird was captured. We measured shrub density by walking perpendicular east-west and north-south transects across the centre of the plot while identifying and counting each individual shrub (defined as a woody plant < 7.6 cm diameter) either intercepted by the observer’s outstretched arms or occurring at a height between the observer’s outstretched arms and knees (adapted from James and Shugart, 1970). All counts were made by two observers of similar height. We also measured ground cover percentage by viewing the ground through an ocular tube at 20 total locations along the two perpendicular transects within the plot (James and Shugart, 1970). Each location was scored as one of the following categories: Japanese barberry, other non-native shrub, native shrub, herbaceous vegetation or ground (i.e. no living vegetation).

### Invertebrate sampling

Leaf-litter arthropod biomass is the standard measure of ovenbird prey abundance (e.g. Burke and Nol, 1998; Mazerolle and Hobson, 2002a; Seewagen *et al*., 2011). To compare leaf-letter arthropod biomass between ovenbird territories with low and high densities of Japanese barberry, we collected 0.04 m^2^ of leaf litter down to the hummus layer at three locations immediately following the capture and processing of each bird—the midpoint of the mist-net and 10 m to the east and west of that point—for a total of 0.12 m^2^ of leaf litter. The leaf litter samples were stored in the field in plastic bags that were inflated with air and kept cool for up to 6 h until being processed at the end of the day. We extracted arthropods from the leaf-litter samples using Berlese funnels that were operated with a 40-watt incandescent lightbulb for 12–18 h. The extracted arthropods were identified to order or family in 2017 as part of a separate study (Clark and Seewagen, 2019) but were not sorted taxonomically in 2018. All arthropods were then oven-dried to a constant mass at 60 °C and weighed to the nearest 0.0001 g. Individuals that were difficult to see without magnification (<1 mm) prior to drying were considered negligible and excluded (Seewagen *et al*., 2011). We considered all taxonomic groups of arthropods to represent potential prey for ovenbirds (Burke and Nol, 1998; Seewagen *et al*., 2011).

### Condition indices

Hb is a protein in red blood cells that is responsible for transporting oxygen from the air sacs of birds to their tissues. Its concentration in the blood is the primary determinant of a bird’s oxygen carrying capacity. High Hb concentrations indicate a high aerobic scope while low concentrations can be indicative of parasitism, disease and nutritional deficiencies (Minais, 2015). TRIG is a plasma metabolite that signals the rate at which dietary and *de novo*-synthesized lipids are being carried through the bloodstream to the adipose tissue for storage (Jenni-Eiermann and Jenni, 1994; Cerasale and Guglielmo, 2006). As such, it is representative of a bird’s feeding activity and used as an indicator of energetic condition and breeding habitat quality (Kilgas *et al*., 2006; Seewagen *et al*., 2015). TPP is representative of the protein content of a bird’s diet (Levielle and Sauberlich, 1961) and commonly used as an indicator of diet quality and nutritional status (Mazerolle and Hobson, 2002b; Schoech and Bowman, 2003; Milenkaya *et al*., 2013). UA is similarly related to dietary protein content and used to indicate diet quality in birds (Smith *et al*., 2007; Seewagen *et al*., 2015). Heterophils and lymphocytes are white blood cells that respectively increase and decrease in response to immune challenges and elevated corticosterone levels. H∶L is therefore considered to be indicative of infection and chronic stress in birds (Suorsa *et al*., 2004; Davis *et al*., 2008; Müller *et al*., 2011). In addition to these haematological condition indices, we also used body mass to compare the energetic condition of ovenbirds between territories with low or high densities of Japanese barberry. We did not adjust body mass to wing length because the two were unrelated (*R*^2^ = 0.004, *P* = 0.635).

### Laboratory analyses

Blood samples were transported to a lab for processing at the end of each day of collection. Smears were prepared with 15 μL of whole blood on glass microscope slides that were then fixed in absolute ethanol, stained (Protocol Hema 3 Stat Pack, Fisher Scientific, Kalamazoo, MI, USA), and air-dried for later measurement of H:L. Another 15 μL of whole blood was used to measure Hb with a HemoPoint H2 point-of-care device (Stanbio Laboratory, Boerne, TX, USA; Georoff *et al.* 2009; Kim *et al.* 2018). The remaining blood from each bird was then centrifuged for 10 min to separate the plasma which was transferred to cryogenic tubes and stored at −80 °C for up to 8 mo until the analysis of TRIG, UA and TPP.

We viewed blood smears under oil immersion at ×1000 magnification and calculated H:L from a total of 100 randomly selected leukocytes (Campbell, 1995). TRIG was measured using a sequential endpoint microplate assay (Sigma Aldrich; Guglielmo *et al*., 2005) and UA was measured by colorimetric endpoint microplate assay (TECO Diagnostics; Smith and McWilliams, 2010). We measured TPP with a Biuret colorimetric endpoint microplate assay (Sigma Total Protein Reagent T1949; Krohn, 2001). We analyzed all samples and standards in duplicate (all CVs < 10%) and ran a chicken plasma pool on each microplate to assess inter-assay variation. Plasma samples were diluted threefold with 0.9% saline solution before all analyses to increase sample volume. When sample volumes were still too small to run all three assays, priority was given to TPP followed by TRIG and UA.

### Statistical analyses

We used the vegetation survey data to assign bird territories with > 4200 Japanese barberry shrubs per ha to a high-density category (*N* = 27) and those with a density of < 2720 Japanese barberry shrubs per ha to a low-density category (*N* = 29). Those with intermediate Japanese barberry densities were omitted from the analyses. We selected these thresholds because they provided nearly balanced sample sizes, allowed most birds to be included in the analyses, and provided a substantial overall difference in Japanese barberry density and ground coverage between the two categories (see Results). There was no qualitative difference in the outcome of our analyses whether we expressed Japanese barberry density this way or as a continuous variable (shrubs per ha); we therefore report only the results of the former.

We ran general linear models (GLMs) with a backward selection procedure (*α* = 0.05) to identify variables that were significantly related to the physiological condition indices. For each physiological condition index, the explanatory variables in the full model included Japanese barberry density (low or high), time of capture (expressed as hours since sunrise), arthropod biomass, age (second-year, after-second-year), body mass and year. Backwards selection is a commonly used method to analyze physiological condition indices in birds (e.g. Guglielmo *et al*., 2005; Seewagen *et al*., 2011, 2015; Oberkircher and Smith Pagano, 2018), but as a general procedure, can have drawbacks and has received criticism in favour of information-theoretic approaches (Whittingham *et al*., 2006). We therefore also calculated Akaike weights of GLMs that were constructed of all possible main effect combinations of our explanatory variables and then summed weights across all models in which a given variable appeared to evaluate the relative importance of that variable (Burnham and Anderson 2002; Symonds and Moussalli, 2011). Variance inflation factors were low for each explanatory variable (all VIF < 1.3), indicating that there was no interference from collinearity in any of our models (Graham 2003; Dormann *et al*. 2013).

**Table 1:** Shrub layer composition of ovenbird breeding territories categorized as having ‘low’ or ‘high’ Japanese barberry density

	Low			High		
	Density (shrubs ha^−1^)	% of shrub community	% of ground cover	Density (shrubs ha^−1^)	% of shrub community	% of ground cover
Japanese barberry	631 ± 1026	8.9 ± 15.4	1.5 ± 3.1	7469 ± 2663	57.2 ± 19.7	30.6 ± 18.1
Other non-native shrubs	156 ± 330	2.8 ± 6.4	0.4 ± 1.4	1798 ± 2895	10.1 ± 14.7	4.8 ± 8.1
Native shrubs	7181 ± 4560	88.4 ± 16.8	20.0 ± 19.6	4594 ± 3475	32.8 ± 19.7	12.8 ± 11.4

Values are means ± SD

**Table 2:** Age ratio and mean (± SD) wing length, body mass and physiological condition indices of ovenbirds breeding in territories with low or high densities of Japanese barberry

	Low	High
Age ratio (second-year/after second-year)	15/13	14/12
Wing length (mm)	77 ± 2 (29)	76 ± 2 (27)
Body mass (g)	19.6 ± 1.0 (29)	19.4 ± 0.9 (27)
Total plasma protein (mg mL^−1^)	34.67 ± 8.63 (27)	34.12 ± 4.95 (23)
Plasma triglyceride (mmol L^−1^)	0.85 ± 0.31 (26)	0.86 ± 0.41 (24)
Plasma uric acid (mmol L^−1^)	1.56 ± 0.70 (23)	1.76 ± 0.78 (22)
Haemoglobin (mmol L^−1^)	8.13 ± 0.94 (19)	8.54 ± 1.25 (17)
Heterophil:lymphocyte	0.47 ± 0.30 (16)	0.48 ± 0.44 (12)

Sample sizes shown in parentheses

We used two-tailed *t* tests to compare arthropod biomass, wing length and body mass between Japanese barberry density categories and a Fisher’s exact test to compare age ratios. TPP, TRIG and UA were log_10_ + 1 transformed, and H:L was square root-transformed to normalize distributions prior to the analyses. All variables met assumptions of equal variance and homogeneous slopes. We ran all statistical analyses using SAS version 9.4 and R version 3.4.3 and accepted significance for all tests when *P* < 0.05.

## Results

### Vegetation composition

Japanese barberry density averaged 7469 plants per ha and represented 57.2% of the shrub community and 30.6% of the ground cover in the high-density territories while averaging only 631 plants per ha and representing only 8.9% of the shrub community and 1.5% of the ground cover in the low-density plots (Table 1). Other non-native shrubs, which included multiflora rose (*Rosa multiflora*), Oriental bittersweet (*Celastrus orbiculatus*) and burning bush (*Euonymus alatus*) were also more abundant in the high-density plots than the low-density plots, but were minimal overall relative to Japanese barberry (Table 1). Native shrubs represented an average of 88.4% of the shrub community in the low-density plots and only 32.8% of the shrub community in the high-density plots (Table 1).

### Arthropods

There was no difference in the biomass of leaf-litter arthropods between ovenbird territories that had low or high densities of Japanese barberry (*t*_1, 47_ = −0.04, *P* = 0.97). Dry mass averaged 0.043 g/0.04 m^2^ (± 0.057 SD) in low-density territories while averaging 0.038 g/0.04 m^2^ (± 0.025 SD) in high-density territories.

### Condition indices

**Figure 1:**
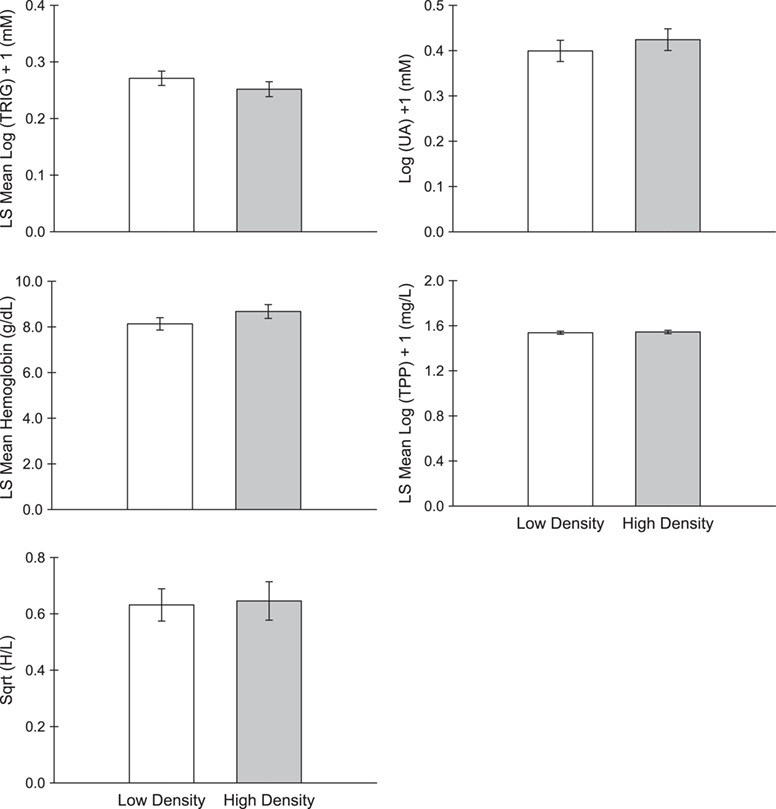
Haematological condition indices of ovenbirds breeding in territories with low or high densities of Japanese barberry. Values are means ± SE for uric acid (UA) and heterophil:lymphocyte (H/L), and least square means ± SE for triglyceride (TRIG), haemoglobin and total plasma protein (TPP), respectively controlling for the effects of capture time, arthropod biomass and body mass

Final sample sizes for each condition index varied depending on blood sample volume, but we were able to collect enough blood to measure at least one haematological condition index in 27 individuals from low Japanese barberry density territories and 24 individuals from high Japanese barberry density territories (Table 2). Variables retained after the backwards selection procedure were few and varied among the different haematological condition indices (Table 3). TRIG had a significant positive relationship with capture time, Hb had a significant positive relationship with arthropod biomass and TPP had a significant positive relationship with body mass. TPP had a negative relationship with arthropod biomass that approached significance (*P* = 0.065). Japanese barberry density, age and year had no significant effect on any of the haematological condition indices (Table 3; Fig. 1).

**Table 3:** Probability values from backwards-selection general linear models of ovenbird physiological condition indices and explanatory variables

	Barberry density	Arthropod biomass	Body mass	Age	Capture time	Year
Triglyceride	0.303	0.676	0.863	0.906	<0.001 (+)	0.761
Total plasma protein	0.518	0.065	0.011 (+)	0.754	0.979	0.767
Uric acid	0.783	0.642	0.982	0.253	0.772	0.402
Haemoglobin	0.199	0.044 (+)	0.274	0.306	0.673	0.220
Heterophil:lymphocyte	1.000	0.447	0.615	0.971	0.412	0.905

Direction of effect for retained variables (*P* < 0.05) indicated by + or −

Summed Akaike weights were in agreement with the results of the backwards selection procedure in that capture time was relatively important for explaining variation in TRIG, arthropod biomass was relatively important for explaining variation in Hb, body mass was relatively important for explaining variation in TPP and neither age nor year were of high relative importance for explaining variation in any of the haematological condition indices. Japanese barberry density also was not relatively important for explaining variation in any haematological condition index, except perhaps TRIG, for which it had a marginally high summed weight of 0.68 behind the highest summed weight of 0.99 for capture time (Table 4). Controlling for the effect of capture time in an analysis of covariance, there was no significant difference in TRIG between Japanese barberry density treatments (*F*_1,47_ = 1.09, *P* = 0.303).

Body mass was similar between both treatment groups (*t*_1, 55_ = 0.75, *P* = 0.46; Table 1). There was also no difference in wing length (*t*_1, 55_ = 1.51, *P* = 0.14) or age ratio (*P* = 1.00) between birds defending territories with a low or high density of Japanese barberry (Table 1).

## Discussion

Invasive plants can impact birds and the quality of their habitat by altering food sources, nesting substrates and other resources that are critical to reproduction and survival (Nelson *et al*., 2017; Narango *et al*., 2018). Japanese barberry is one of the most widespread non-native woody plants in many forested areas of the northeastern USA, and yet almost nothing is known about its effects on birds or other wildlife. Here, we investigated for the first time the relationship between Japanese barberry and the prey abundance and associated physiological condition of a North American forest-breeding bird. We found no difference in the biomass of leaf-litter arthropods, the main food source of ovenbirds, between heavily invaded forest and forest patches that had minimal to no Japanese barberry. This may largely explain why we also found no indication that ovenbirds whose nesting territories were dominated by Japanese barberry were in poorer physiological condition than those whose territories were far less invaded by Japanese barberry. Body mass and all five of our haematological condition indices were unrelated to the density of Japanese barberry in their breeding territories. Our findings suggest that at the densities observed in our study site, Japanese barberry does not affect habitat quality for breeding male ovenbirds in a way that affects their food abundance or physiological condition.

Non-native plants have been widely shown to decrease the richness and diversity of aboveground and belowground arthropod communities (Litt *et al*., 2014), but arthropod biomass is not always affected by these shifts in community structure (Tanner *et al*., 2013). This appears to be true of Japanese barberry as well, which we have shown to be associated with lower arthropod richness and abundance, but not a change in arthropod biomass (Clark and Seewagen, 2019). In such cases, invertivores may not be affected by the changes in arthropod community composition caused by non-native plants, provided they are not specialized to feed on any particular taxonomic groups. Ovenbirds are considered to be dietary generalists that feed on a variety of leaf-litter arthropods (Stenger, 1958; Holmes and Robinson, 1988) roughly in proportion to availability (Stenger, 1958). However, their dietary breadth does not necessarily mean that they do not partly depend on certain arthropod taxa to meet nutritional needs for themselves, their mates or their nestlings. It is possible that changes in the relative abundance of certain groups in the arthropod community caused by Japanese barberry could negatively impact ovenbirds without reducing the total biomass of food available to them. Ants, for example, are a major component of the ovenbird’s diet (Stenger, 1958; Holmes and Robinson, 1988; Strong and Sherry, 2000) and are only one-fifth as abundant in areas with a high density of Japanese barberry as they are in areas with comparatively little Japanese barberry (Clark and Seewagen, 2019). It is unknown whether having to shift towards other arthropods to compensate for a reduced abundance of ants or other taxonomic groups would have any consequences. Our haematological condition indices indicated that there were no differences in general nutritional status or dietary energy or protein content between ovenbirds inhabiting areas with low and high densities of Japanese barberry. However, they do not provide detailed information about many other aspects of diet quality that are important to birds, such as amino acid and highly unsaturated fatty acid composition (Ramsay and Houston, 2003; Twining *et al*., 2016a,b), and antioxidant content (Costantini, 2008). How changes in arthropod community structure driven by non-native plants affect the composition and quality of the diet of invertivores is an open and interesting subject for future investigation.

Haematological condition indices have been measured in breeding ovenbirds in two previous studies of which we are aware, to evaluate individual-level impacts of forest fragmentation from agriculture (Mazerolle and Hobson, 2002b) and exurban housing development (Seewagen *et al*., 2015). Although forest fragmentation has been widely found to reduce ovenbird pairing success and productivity (Porneluzi *et al*., 2011), neither study found evidence of a physiological impact. The mean TPP levels and H:L ratios of the ovenbirds nesting among high densities of Japanese barberry in our study were comparable to those of ovenbirds breeding in reference forests of high quality in Saskatchewan, Canada (Mazerolle and Hobson 2002b). In addition, their TPP and UA levels and H:L ratios were superior on average to those of male ovenbirds breeding in both fragmented and intact, high-quality reference forests in the Adirondack Mountains of New York, USA (Seewagen *et al*., 2015). The general similarity of these haematological parameters between ovenbirds nesting in areas heavily invaded by Japanese barberry and those nesting in the reference forests used in these other studies further suggests that Japanese barberry does not impact habitat quality for breeding male ovenbirds in a way that affects their physiological condition.

**Table 4:** Summed Akaike weights indicating relative importance of variables for explaining variation in physiological condition indices of breeding male ovenbirds

	Barberry density	Arthropod biomass	Body mass	Age	Capture time	Year
Triglyceride	0.68	0.23	0.23	0.28	0.99	0.23
Total plasma protein	0.23	0.33	0.85	0.24	0.23	0.36
Uric acid	0.29	0.49	0.24	0.26	0.30	0.44
Haemoglobin	0.41	0.83	0.29	0.32	0.28	0.34
Heterophil:lymphocyte	0.23	0.41	0.25	0.25	0.26	0.24

Ovenbird body size and age can also provide information about habitat quality, as some studies have observed higher-quality breeding habitat to be occupied by larger and older males (Bayne and Hobson, 2002; Mazerolle and Hobson, 2002b; but see Burke and Nol, 2001). This suggests that the habitat that is in greatest demand and most difficult to defend might be acquired by only the most dominant individuals. We did not find any such pattern in body size or age ratio between our two treatment types to indicate a difference in habitat quality or demand. The areas heavily invaded by Japanese barberry were not occupied by smaller ovenbirds or more first-time breeders than areas that were relatively free of Japanese barberry.

We are aware of only three previous studies to investigate impacts of Japanese barberry to North American birds, and they also found birds inhabiting invaded areas to be unaffected. Veeries (*Catharus fuscescens*) that built nests within Japanese barberry shrubs in a forest in New York State were found to have similar nesting success as those nesting in native vegetation and greater nesting success than those nesting on the ground, likely because of the visual concealment and protection from predators provided by Japanese barberry (Schmidt *et al*., 2005; Meyer *et al*., 2015). They also appeared to preferentially nest in Japanese barberry over native plants and nested more often in habitat patches that were heavily invaded (Meyer *et al*., 2015). Schlossberg and King (2010) similarly found a suite of shrubland bird species in Massachusetts to prefer and experience comparable or greater nesting success in non-native shrubs, including Japanese barberry, over native shrubs.

One possibility that is worth consideration is that Japanese barberry provides benefits to some plant and animal species in mature forests, like our study site, that experience heavy browsing pressure from white-tailed deer. Many such forests lack the dense understory that is normally created by a mix of native shrubs and regenerating trees (Habeck and Schultz, 2015). Because it is largely unpalatable to deer, Japanese barberry often provides a dense understory and adds structural heterogeneity to woodlands where these features would otherwise be largely lacking as a result of over-browsing. The forest shrub layer is important to many wildlife species for shelter, predator concealment, nesting substrates and other resources, and it is possible that by growing in areas where deer densities are high, Japanese barberry provides some beneficial services as a surrogate for over-browsed native plants. We previously showed that arthropod abundance is lower on Japanese barberry than on native shrubs, but on a per-shrub basis (Clark and Seewagen, 2019). The greater overall density of shrubs in invaded areas than in areas with little to no Japanese barberry may compensate for this difference and actually provide greater food abundance for invertivores per unit area. Additionally, dense and thorny shrubs like Japanese barberry sometimes act as barriers to deer and thereby protect coexisting native plants from browsing (Harmer *et al*., 2010; Jensen *et al*. 2012; Salek *et al*., 2019). This may explain why the territories with high Japanese barberry density in our study site maintained nearly two-thirds the average density of native shrubs as the territories with low Japanese barberry density. Altogether, it is possible that the substantially greater total density of native and non-native shrubs in the invaded areas of the forest increases resource availability to ovenbirds and other understory/ground-dwelling wildlife.

Our results point towards the importance of investigating and understanding the effects of non-native plant invasions on native wildlife before taking management action (Schirmel *et al.* 2016; Nelson *et al*., 2017). The relationships between non-native, invasive plants and birds are likely to be complex and species-specific, and should not always be presumed to be negative (Nelson *et al*., 2017). We did not find any indication of a negative effect of Japanese barberry on the prey abundance or physiological condition of breeding male ovenbirds that would warrant aggressive management on behalf of this species. However, we recognize that our study was limited to only a single forest and two breeding seasons, and we cannot eliminate the possibility of differing effects of Japanese barberry in other sites or years that have different environmental conditions. We also caution that adult female and nestling ovenbirds in barberry-invaded habitat could experience impacts to their condition while adult males do not. Most importantly, we examined only one of the many bird species that breed in Japanese barberry-invaded forests of the eastern USA and did not investigate any other aspects of habitat quality that could be negatively affected. We encourage future research on additional bird species and the effects of Japanese barberry on other important factors, such as diet composition and quality, pairing success, nestling quality, nest success and post-fledging survival. This would allow for more science-based decision-making by land managers and conservation practitioners who are grappling with the invasion of northeastern US forests by Japanese barberry. Combating Japanese barberry invasions on a large scale is extremely labour-intensive and costly (Ward *et al*., 2013), and the effects of this plant on birds and other wildlife should be better understood before allocating limited conservation resources to its control.

## Funding

This work was supported by operating funds of the Great Hollow Nature Preserve & Ecological Research Center, faculty research funds from the Rochester Institute of Technology Thomas H. Gosnell School of Life Sciences and a grant from the Connecticut Ornithological Association.
